# CHI3L1 (Chitinase 3 Like 1) upregulation is associated with macrophage signatures in esophageal cancer

**DOI:** 10.1080/21655979.2021.1974654

**Published:** 2021-10-06

**Authors:** Jing Huang, Zhenlin Gu, Yingying Xu, Lei Jiang, Weiguo Zhu, Wanwei Wang

**Affiliations:** a Department of Radiation Oncology, The Affiliated Huaian No.1 People’s Hospital of Nanjing Medical University, Huaian, China; b Department of Vascular Surgery, The Affiliated Huaian No.1 People’s Hospital of Nanjing Medical University, Huaian, China

**Keywords:** Esophageal cancer, CHI3L1, macrophage, M2 polarization, tumor microenvironment (TME)

## Abstract

Chitinase-3 like-protein-1 (CHI3L1) has been found to be overexpressed in many cancers and increased CHI3L1 level in serum seems to correlate with a poor prognosis in patients with metastatic cancer. However, the expression of CHI3L1 and its potential role in esophageal cancer remains unclear. We retrieved publicly available RNA-seq datasets of esophageal cancer tissues and normal esophageal tissues. We analyzed the correlation between CHI3L1 expression with different clinical parameters (such as T stages, N stage, response to treatment and tumor residues after treatment), the relationship between CHI3L1 expression level and prognosis, and the relationship between CHI3L1 expression and different immune cell signatures in esophageal cancer tissues. A transgenic mouse model of esophageal carcinoma was used to validate CHI3L1 expression and its association with macrophage signature gene expression. The effect of recombinant CHI3L1 on macrophage polarization was assessed in cell model. We showed the upregulation of CHI3L1 in esophageal cancer tissues in comparison to normal esophageal tissues, and its upregulation was positively associated with tumor size. The analysis of immunological signatures and CHI3L1 expression indicated that CHI3L1 level was highly correlated with increased expression of macrophage signature genes in esophageal tumor tissues. CHI3L1 was also upregulated in the esophagus dysplasia tissues in a transgenic mouse model. Recombinant CHI3L1 treatment favored M2 gene expression in LPS-stimulated RAW 264.7 macrophage cell line. CHI3L1 overexpression may favor macrophage recruitment in esophageal tumor tissues. Future studies are needed to delineate the mechanisms of CHI3L1-mediated macrophage recruitment and polarization in tumor tissues.

## Introduction

Oral cancer is one of the most common cancers in the head and neck, with about 405,000 diagnosed cases every year [1]. Among them, esophageal cancer is the eighth most common cancer and the sixth leading cause of death from cancer worldwide [2]. The escalating incidence of EC with its recurrence has imposed tremendous pressures on the health-care system [3,4]. Despite of recent advancement of cancer treatment, EC remains as a great life-threatening cancer with an unfavorable prognosis, with an overall 5-year overall survival rate of 18% [5]. Although early diagnosis favors a better treatment outcome [6], many risk factors contribute to the development of EC and the lack of effective diagnosis biomarker hinder the population-wide screening for early detection [2].

Tumor microenvironment (TME) is a unique microsystem comprising a variety of cell types to support unrolled tumor growth and facilitate immune evasion. Immune cells are the major components recruited to TME, among which regulatory T cells (Tregs) and tumor-associated macrophages (TAMs) are two major types of immune cells conferring immunosuppression [7]. Tregs are preferentially enriched in TME and its unique metabolic adaptation enables its survival and the establishment of suppression on other immune cells [8]. The recruitment of macrophages into TME leads to M2-like polarization in TAMs, which promotes the immunosuppression, tumor metastasis as well as chemoresistance [9]. Targeting tumor-infiltrating Tregs and repolarization of TAM are attractive strategies to remodel the immune responsiveness in TME to serve as a combinatory treatment with chemotherapy or augment the efficacy of immunotherapy [10].

Chitinase-3 like-protein-1 (CHI3L1) is one of the chitinase-like proteins without chitinase enzymatic activity. HI3L1 is produced by a variety of cells, including macrophages, fibroblast-like cells, hepatic stellate cells, endothelial cells and cancer cells [11]. CHI3L1 can be induced by a multitude of signals, including extracellular matrix (ECM) remodeling, growth factors, cytokines, cellular stress and chemical perturbation [12–14]. It is well known that CHI3L1 is implicated in a wide spectrum of biological regulations such as pathogen defense, tissue repair, ECM remodeling, inflammatory responses, and macrophage differentiation [11,15]. Interestingly, CHI3L1 has been reported to be overexpressed in many cancers and increased CHI3L1 level in serum seems to correlate with a poor prognosis in patients with metastatic cancer [16,17]. However, the expression of CHI3L1 and its potential role in esophageal cancer remains to be elucidated.

In this study, we aim to investigate the expression level of CHI3L1 in esophageal cancer and its correlation with immune cell types. We surveyed publicly available RNA-seq datasets which comprised esophageal cancer tissues and normal esophageal tissues. Our analysis showed the upregulation of CHI3L1 in esophageal squamous cell carcinomas in comparison to normal esophageal tissues. CHI3L1 expression level was positively associated with tumor size, and elevated level of CHI3L1 in esophageal cancer tissues caused major changes ECM and cell junction gene programs. Specifically, analysis of immunological signatures and CHI3L1 expression indicated that CHI3L1 level was highly correlated with increased expression of macrophage signature genes in esophageal carcinoma. CHI3L1 was also upregulated in the esophagus dysplasia tissues in a transgenic mouse model and was associated with the changes in macrophage polarization marker genes. Collectively, our data indicates that the elevated level of CHI3L1 expression in esophageal cancer may favor the recruitment of macrophage in TME to support the progression of esophageal cancer.

## Materials and methods

### Esophageal carcinoma data retrieval and analysis

We retrieved the RNA-seq data from TCGA (https://portal.gdc.cancer.gov/) ESCA (esophageal cancer) project level 3 containing HTSeq-FPKM format RNA-seq data. We selected the data of esophageal squamous cell carcinomas (162) and 11 normal esophageal tissues for the comparative analysis of CHI3L1 expression. In addition, the RNA-seq data of 555 normal esophagus – mucosa samples in GTEx Portal database (https://gtexportal.org/home/) was analyzed against the data of 162 esophageal cancer samples in TGCA database. Differential gene expression analysis was performed using raw counts data by Deseq2 package [18]. Genes with fold change >1.5 or <0.75 and adjusted *p* < 0.05 were considered as differentially expressed. For the display of normalized expression RNAs-seq data, FPKM (Fragments Per Kilobase per Million) is converted into TPM (transcripts per million reads) format with log2 conversion.

Receiver operating characteristic (ROC) analysis to evaluate the effectiveness of CHI3L1 expression levels in distinguishing esophageal cancer tissues from non-tumor tissues. RNA-seq data in ESCA (esophageal cancer) project from TCGA database were used (including all esophageal cancer tissues from non-tumor tissues). pROC package and ggplot2 package were used for ROC analysis and visualization. Statistical analysis was performed using R (version 4.0.3; R Foundation for Statistical Computing, Vienna, Austria).

To analyze the correlation between CHI3L1 expression with different clinical parameters, such as T stages, N stage, response to treatment and tumor residues after treatment, ggplot2 package was used for visualization and statistical analyses was performed with Mann–Whitney U test.

To analyze the relationship between CHI3L1 expression level and prognosis, the median of CHI3L1 expression level was used as the cutoff to divide esophageal cancer patients into low and high-expression group. Kaplan Meier curve analysis was performed using survminer package (for visualization), survival package (for statistical analysis of survival data).

To investigate what gene programs are dysregulated between high CHI3L1 expression and low-expression groups, Gene Ontology Enrichment analysis was performed using DAVID bioinformatics online tool (https://david.ncifcrf.gov/). Gene Set Enrichment Analysis (GSEA) was performed using the clusterProfiler package [19]. Gene sets c2.cp.v7.2.symbols.gmt in MSigDB Collections (http://www.gsea-msigdb.org/gsea/msigdb/collections.jsp) was used as references. The conditions of False discovery rate (FDR) <0.25 and *p*.adjust <0.05 were considered to be significant enrichment.

To find the relationship between CHI3L1 expression and different immune cell signatures in esophageal cancer tissues, RNA-seq data in ESCA project (162 esophageal cancer tissues) were subject to analysis using R package: GSVA, Immune cell algorithm: ssGSEA (GSVA package built-in algorithm) [20]. The signature genes of 24 immune cell types were retrieved from [21].

### Cell culture and treatment

Murine macrophage/monocyte cell line RAW 264.7 was (purchased from the Cell Bank of the Chinese Academy of Sciences, China) was maintained in RPMI-1640 medium (Gibco BRL, New York, USA) supplemented with 10% fetal bovine serum (FBS), 100 mg/mL streptomycin and 100 U/mL penicillin underhumidified culture conditions (37°C and 5% CO2 incubator). For stimulation, 10 µg/ml Lipopolysaccharide (LPS) (00–4976-03, Invitrogen, USA) was applied for 24 hours. Mouse YKL-40/CHI3L1 recombinant protein was purchased from Sino Biological (50,929-M08H, Japan) and reconstituted in distilled water. Recombinant CHI3L1 was used at a final concertation of 200 ng/ml.

### RNA extraction and qPCR

To evaluate mRNA transcript level, total RNA was extracted from tissues or cultured cells using Trizol reagent (15,596,026, Invitrogen, USA) according to the manufacturer’s protocol. 5 µg total RNA was reverse transcribed to cDNA using a PrimeScript RT Reagent Kit (RR037B, Takara, Japan) according to the manufacturer’s instructions. Quantitative real-time qPCR was performed using a StepOnePlus™ System (Applied Biosystems, Foster City, CA, USA) with SYBR™ Green PCR Master Mix (4,309,155, Thermo Fisher Scientific, USA). Glyceraldehyde-3-phosphate dehydrogenase (GAPDH) gene was used as an endogenous control to normalize the expression level of the target gene in different samples. The fold changes were calculated using a relative quantification method (2− ΔΔCt). qRT-PCR assays were performed independently for at least three times. The primer sequences used in this study are listed as follows: GAPDH (Forward: CGGAGTCAACGGATTTGGTCGTAT; Reverse: AGCCTTCTCCATGGTGGTGAAGAC).

CHI3l1 (Forward: AGACGCCATCCAACCTTTCC; Reverse: GTTCGACTCGTCATTCCACTC).

IL1β (Forward: GCAACTGTTCCTGAACTCAACT; Reverse: ATCTTTTGGGGTCCGTCAACT).

TNFα (Forward: CTGAACTTCGGGGTGATCGG; Reverse: GGCTTGTCACTCGAATTTTGAGA).

TGFβ1 (Forward: CTTCAATACGTCAGACATTCGGG; Reverse: GTAACGCCAGGAATTGTTGCTA).

MRC1 (Forward: CTCTGTTCAGCTATTGGACGC; Reverse: CGGAATTTCTGGGATTCAGCTTC).

### Transgenic mouse model for esophageal carcinoma

A transgenic mouse model for Esophageal carcinoma was previously established by overexpressing human cyclin D1 cDNA under the Epstein-Barr virus ED-L2 promoter [22]. This transgenic muse line was prepared and purchased from KeyGen BioTECH (Jiangsu, China). Mice were raised under the pathogen-free condition. Developed dysplasia (a prominent precursor to carcinoma) were observed at esophagus and forestomach at the age of 14–16 months. Mice with developed esophagus dysplasia were sacrificed at the age of 16 months. Carbon dioxide was used for euthanizing. Briefly, a euthanizing chamber was connected to a carbon dioxide cylinder and the flow rate was adjusted to displace 20% of the cage volume per minute. Mice were placed into the euthanizing chamber for 10 mins until no movement was observed. Death was assured by cervical dislocation. After dissection, esophagus dysplasia tissues and adjacent normal tissues were collected and snap-frozen in liquid nitrogen before further analysis.

## Results

In this study, we surveyed publicly available RNA-seq datasets containing esophageal cancer tissues and normal esophageal tissues. We found the upregulation of CHI3L1 in esophageal squamous cell carcinomas in comparison to normal esophageal tissues. Specifically, analysis of immunological signatures and CHI3L1 expression indicated that CHI3L1 level was highly correlated with increased expression of macrophage signature genes in esophageal carcinoma. CHI3L1 was also upregulated in the esophagus dysplasia tissues in a transgenic mouse model and was associated with the changes in macrophage polarization marker genes. Collectively, our data indicates that the elevated level of CHI3L1 expression in esophageal cancer may favor the recruitment of macrophage in TME to support the progression of esophageal cancer.

### CHI3L1 is upregulated in esophageal carcinoma

**Figure 1.**
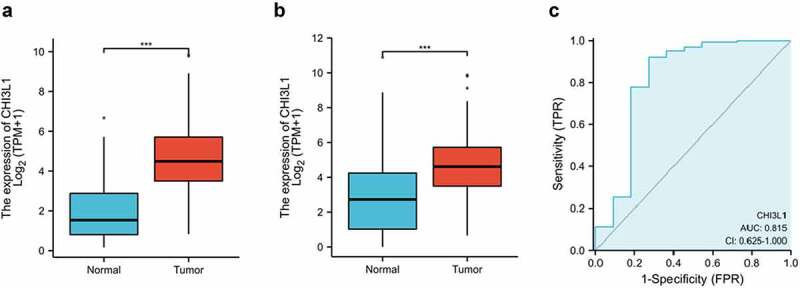
CHI3L1 is upregulated in esophageal carcinoma. (a) The expression level of CHI3L1 in 162 esophageal cancer tissues and 11 normal esophageal tissues. Data from TCGA ESCA (esophageal cancer) project. (b) The expression level of CHI3L1 in 162 esophageal cancer tissues from TCGA and the 555 Esophagus – Mucosa samples in GTEx Portal database. (c). Receiver Operating Characteristics (ROC) curve of CHI3L1 expression level for predicting esophageal cancer. Analysis was based on the data from 162 esophageal cancer tissues and 11 normal esophageal tissues. AUC, area under the curve; CI, confidence interval; TPR, true positive rate; FPR, and false positive rate. Statistics in (a-b): Mann–Whitney U test. *** *p* < 0.001

To investigate the expression level of CHI3L1 in esophageal carcinoma, we retrieved the RNA-seq data in ESCA (esophageal cancer) project from TCGA database (The Cancer Genome Atlas, https://portal.gdc.cancer.gov/). We analyzed the expression level of CHI3L1 in 162 esophageal squamous cell carcinomas and 11 normal esophageal tissues, and found that CHI3L1 was highly expressed in esophageal cancer tissues (P < 0.001, Figure 1a). In addition, the RNA-seq data of 555 normal esophagus – mucosa samples in GTEx Portal database (https://gtexportal.org/home/) processed uniformly in the TPM format was analyzed against the data of 162 esophageal cancer samples in TGCA database. We also found that CHI3L1 was significantly upregulated in esophageal cancer tissue samples (P < 0.001, Figure 1b). We further applied receiver operating characteristic (ROC) curves to analyze the effectiveness of CHI3L1 expression levels in distinguishing esophageal cancer tissues from non-tumor tissues. ROC curve demonstrated that the variable CHI3L1 expression level could predict the tumor samples with certain accuracy (AUC = 0.815, CI = 0.625–1.000) (Figure 1c). Together, the above data imply that CHI3L1 is a potential biomarker for esophageal carcinoma.

### CHI3L1 expression level informs the primary tumor size of esophageal carcinoma

**Figure 2.**
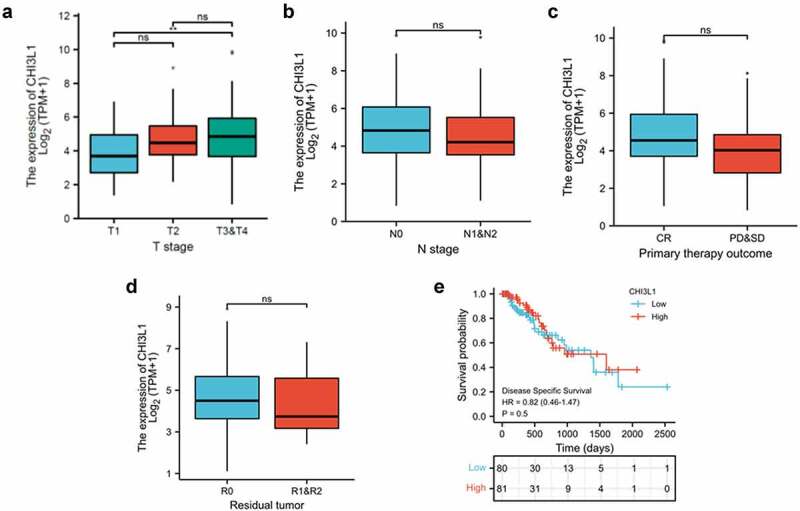
CHI3L1 expression level informs the primary tumor size of esophageal carcinoma. (a) Comparison of the relationship between CHI3L1 expression level and the T staging of esophageal cancer patients in the TCGA database. (b) Comparison of the relationship between the expression of CHI3L1 and the N stage of esophageal cancer. (c) Comparison of the relationship between the expression of CHI3L1 and the initial treatment results of esophageal cancer patients. (d) Comparison of the relationship between CHI3L1 expression and tumor residues in esophageal cancer patients. (e) The expression level of CHI3L1 and the prognosis in patients with esophageal cancer. The median of CHI3L1 in all patients was used as the cutoff for low and high expression. Statistics in (a-d): Mann–Whitney U test. ** p < 0.05

We next attempted to assess whether the CHI3L1 correlates with different clinical parameter of tumor, such as T stages (Tumor size and location, the ligher the number after T, the larger tumor or the more it has grown into nearby tissues), N stage (lymph node metastasis, higher the number after the N indicates the more metastasis in lymph nodes), response to treatment (Complete Response (CR). progressive disease (PD), and stable disease (SD)) and tumor residues after treatment (R0 corresponds to resection for cure or complete remission. R1 to microscopic residual tumor and R2 to macroscopic residual tumor). We found that a significantly higher CHI3L1 expression level was associated with T3&T4 tumor as compared to T1 tumor (Figure 2a, T1 vs T3&T4). However, no significant changes of CHI3L1 expression was observed in tumors with different N stages, response to treatment and tumor residues (Figure 2b–d). We also used the median of CHI3L1 expression level to divide the patient into low- and high-expression group. However, Kaplan Meier curve analysis revealed no significant changes in the survival probability between these two groups (Figure 2e). Therefore, CHI3L1 expression level correlates with primary tumor size, but seems not associated with lymph node metastasis, treatment response and the prognosis.

### High CHI3L1 expression level is enriched with ECM and cell junction biological processes

**Figure 3.**
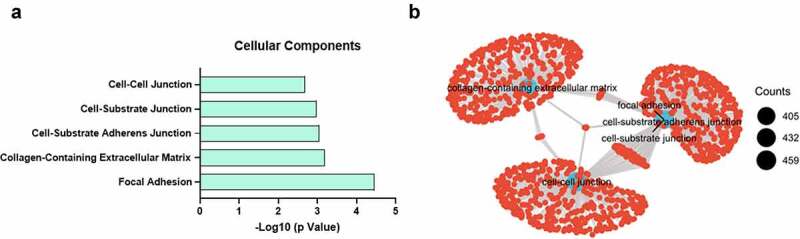
High CHI3L1 expression level is enriched with ECM and cell adhesion biological processes. (a) The median of CHI3L1 in all patient samples was used as the cutoff to divide 162 esophageal cancer to low and high expression group. The differentially expressed genes between the two groups were subject to Gene Ontology (GO) enrichment analysis. The enriched cellular components (CC) were shown with their adjusted p value (FDR). (b) The differentially expressed genes were subjected to Gene Set Enrichment Analysis (GSEA) and the over-represented gene clusters/sets in the high expression group (with p.adj<0.05) were visualized by clusterProfiler. The size of circle represents the number of gene counts

We next asked the question of what gene programs are dysregulated between high CHI3L1 expression and low-expression groups. The median of CHI3L1 expression level was used as the cutoff to divide the patient samples into low- and high-expression group. Differential gene expression analysis was performed using raw counts data by Deseq2 package [18]. Differentially expressed genes (fold change > 1.5, adjusted p < 0.05) were subject to KEGG pathway enrichment analysis using DAVID bioinformatics online tool [23]. We found that cellular components regarding cell–cell interactions such as focal adhesion, extracellular matrix and adherens junction were highly enriched in the differentially expressed genes (Figure 3a). We further applied Gene Set Enrichment Analysis (GSEA) to find the enriched genes sets [24], and performed connectivity analysis by clusterProfiler [19] to visualize the network of the significantly enriched gene sets. Consistently, gene sets related to focal adhesion, extracellular matrix and cell junction were the key hub of the network, indicating they are the key gene programs altered between CHI3L1 low- and high-expression groups (Figure 3b).

### CHI3L1 expression is highly correlated with the infiltration of Macrophage in esophageal carcinoma

**Figure 4.**
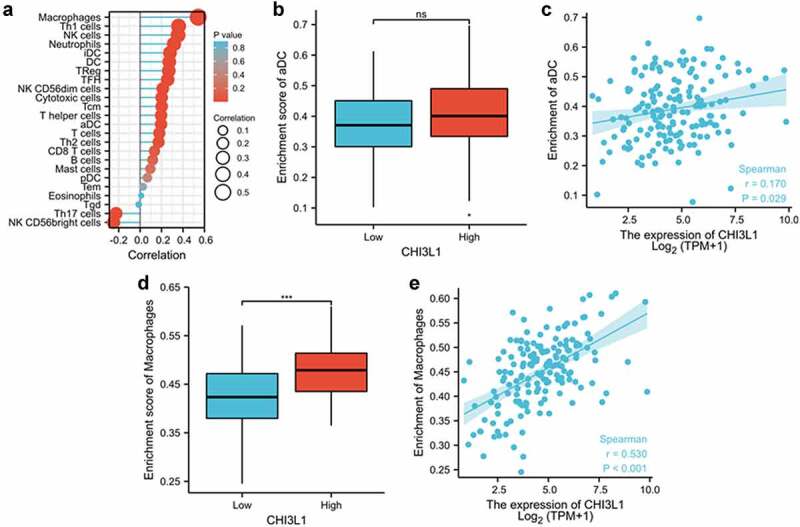
CHI3L1 expression is highly correlated with the infiltration of Macrophage in esophageal carcinoma. (a) The forest plot showed the correlation between the expression level of CHI3L1 and 24 types of immune cells. The size of the dot represents the absolute value of Spearman correlation coefficient r, and the Wilcoxon rank sum test was used to analyze the difference in tumor infiltration between CHI3L1 high expression group and the low expression group. aDC [activated DC], DC [dendritic cells]; iDC [immature DC]; NK [natural killer]; pDC [Plasmacytoid DC]; Tcm [T central memory]; Tem [T effector memory]; Tfh [T follicular helper]; Tgd [T gamma delta]; Treg [regulatory T cells]. (b) Wilcoxon rank sum test was used to analyze the difference of activate DC infiltration level between high CHI3L1 expression group and the low expression group. The enrichment score was calculated based on the normalized expression levels of signature genes of activate DC between CHI3L1 low and high expression group. (c) Spearman correlation method was used to detect the correlation between CHI3L1 expression and DC cell enrichment. (d) Wilcoxon rank sum test was used to analyze the difference of macrophage infiltration level between high CHI3L1 expression group and the low expression group. (e) Spearman correlation method was used to detect the correlation between CHI3L1 expression and macrophage cell enrichment. Statistics in (b, d): Mann–Whitney U test. *** p < 0.001; in (c, e): Spearman’s rank correlation coefficient analysis

Since CHI3L1 has been reported to regulate functions of different immune cells [25,26], which are important components of tumor microenvironment, we further assessed whether CHI3L1 expression level correlated with the infiltration of different immune cells. RNA-seq data in ESCA (esophageal cancer) project (162 esophageal cancer tissues) were used for the correlation analysis between immune cell signatures and CHI3L1 expression level using ssGSEA immune cell algorithm (GSVA package built-in algorithm) [20]. The signatures for 24 immune cell type was retrieved from [21]. The analysis assesses the correlation between the expression level of the immune signatures and the target gene (CHI3L1). Forest plot showed the correlation between the expression level of CHI3L1 and the signature genes of 24 immune cells (Figure 4a). Macrophages, Th1 cells, NK cells, neutrophils and dendritic cells were among the top raked cell types associated with high level of CHI3L1 expression. We selected a weak correlation cell type (activated Dendritic cells) and the strong correlation cell type (Macrophage) for detailed analysis. The enrichment score (normalized expression level of signature genes) of aDC (activated Dendritic cells) showed only a weak correlation with CHI3L1 expression level (r = 0.170, Figure 4c), and Wilcoxon rank sum test revealed no significant difference of aDC enrichment score between CHI3L1 low- and high-expression group (Figure 4b). However, for the macrophages with a strong correlation with CHI3L1 expression level (r = 0.530, Figure 4e), its signature genes (macrophage enrichment score) were significantly upregulated in the CHI3L1 high-expression group (Figure 4d). Together, these data suggest that macrophage represents as a dominant type of innate immune cells infiltrated in the esophageal carcinoma with high CHI3L1 expression.

### CHI3L1 expression is elevated in esophageal carcinoma of a mouse model, and is associated with macrophage polarization markers

**Figure 5.**
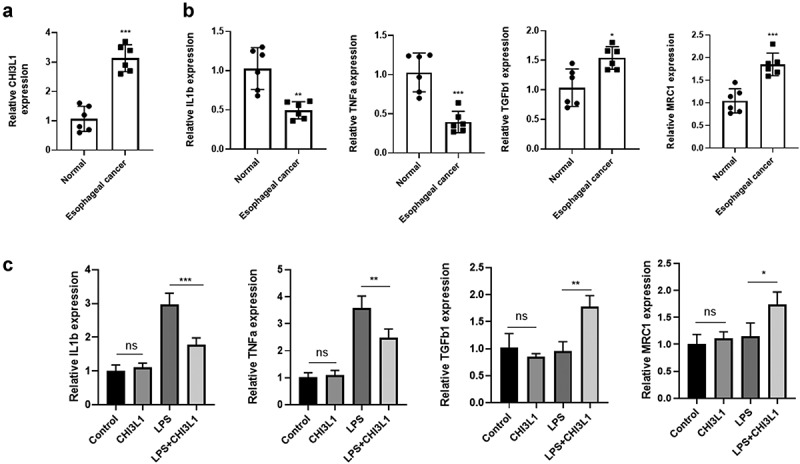
CHI3L1 expression is elevated in esophageal carcinoma of a mouse model, and is associated with macrophage polarization markers. (a). Normal esophageal tissues (n = 6) and esophageal tumor tissues (n = 6) in a transgenic mouse model were collected. The expression level of CHI3L1 was quantified by qPCR. (b). The M1 macrophage markers (IL1β and TNFα) and M2 markers (TGFβ1 and MRC1) were analyzed by qPCR in normal esophageal tissues (n = 6) and esophageal tumor tissues (n = 6). (c). Murine RAW 264.7 macrophage cells were treated with LPS, recombinant CHI3L1 or both for 24 hours, and the expression level of IL1β, TNFα, GFβ1 and MRC1 was quantified by qPCR (n = 3 independent experiments). Statistics: (A_B): unpaired students’ t-test, (C) one way-ANOVA with Tukey’s post-hoc test. * *p* < 0.05; ** *p* < 0.01; *** *p* < 0.001

To validate the finding that CHI3L1 is upregulated in esophageal carcinoma and is associated with macrophage function, we analyzed CHI3L1 expression in a transgenic mouse model of esophageal carcinoma [22]. Esophageal carcinoma tissue (n = 6) and para-cancerous normal tissues (n = 6) were collected and qPCR analysis showed a significant upregulation of CHI3L1 in the tumor tissues (Figure 5a). Since CHI3L1 has been showed to regulate the inflammatory properties of macrophages [11], we next quantified the relative expression levels of M1-macrohage and M2 Macrophage associated genes. The expression of proinflammatory M1 macrophage genes such as IL1β and TNFα were significantly downregulated in esophageal carcinoma tissues, while M2 marker genes TGFβ1 and MRC1 were upregulated. We next used the murine RAW 264.7 macrophage cell line to investigate the effect of recombinant CHI3L1 on its polarization under LPS stimulation. We found that under resting stage (without LPS stimulation), CHI3L1 showed no effect on the expression of M1 and M2 genes (Figure C). However, the CHI3L1 significantly suppressed the upregulation of IL1β and TNFα induced by LPS. Interestingly, CHI3L1 treatment also led to increased expression of M2 genes TGFβ1 and MRC1 after LPS stimulation. Together, the above data indicates that the upregulation of CHI3L1 is correlated with a polarized phenotype of macrophage.

## Discussions

A growing body of evidence substantiates the roles of CHI3L1 in cancer development. It has been found to be upregulated in many types of cancers including, acute myeloid leukemia [27], lung cancer [28], colorectal carcinoma [29], prostate carcinoma [30] and breast cancer [31]. Importantly, the elevated level of CHI3L1 is often correlated with a poor prognosis and with aggressive and metastatic features in tumors [27,29–31]. A previous study also demonstrated that serum CHI3L1 may represent a diagnostic biomarker for esophageal squamous cell carcinoma patients [32]. In accordance with these findings, our analysis revealed an upregulated level of CHI3L1 in esophageal tumor tissues. Although we did not find a significant correlation between CHI3L1 level in esophageal tumor tissue and the survival of the patients with esophageal cancer or the lymph node metastasis, a high level of CHI3L1 level seems to inform a relative larger tumor size. The ROC analysis also indicates that CHI3L1 level could predict esophageal cancer with certain accuracy.

Our study also revealed that increased expression level of CHI3L1 seems to positively correlate with the signature genes of macrophages, Th1 cells, NK cells, neutrophils and dendritic cells in esophageal tumor tissues, indicating an enhanced infiltration of these cells into high CHI3L1 expression tumors. Among them, macrophages showed the strongest positive correlation with CHI3L1 expression. Tumor-associated macrophage (TAM) constitutes the major immune component in tumor microenvironment to establish the immunosuppression [33]. Consistent with our finding, a previous study suggests that CHI3L1 promotes macrophage recruitment in colorectal cancer [34]. Melanoma Associated CHI3L1 was also reported to promote immune cell recruitment [35]. The interaction of CHI3L1 with the extracellular matrix may trigger the remodeling of extracellular matrix and the inflammatory activation of endothelial cells, which could increase the abundance of various cytokines such as CC-chemokine ligand2 (CCL2) for immune cell recruitment [35]. In addition, recombinant CHI3L1 could also function to release the heparan sulfate-bound vascular endothelial growth factor A (VEGF-A), which induces local angiogenesis [34,35]. The augmented neovascularization may also facilitate the recruitment of immune cells such as macrophages.

Apart from the immune cell recruitment, CHI3L1 has been reported to modulate immune phenotypes of different immune cells, such as helper T cell differentiation and macrophage polarization. For instance, in lung cancer mouse model CHI3L1-deficient T cells are prone to differentiating into Th1 cells with enhanced anti-tumor activity [36]. A recent study reveals that stimulation of macrophage with recombinant CHI3L1 induced the transition to M2 phenotype [37]. In addition, in a food allergy mouse model, CHI3L1 expression is increased in the intestinal macrophages, which leads to M2-like macrophage polarization [38]. CHI3L1 has been recently reported to modulate M2-like phenotype of tumor-associated macrophages, which contributes to immune suppression in glioblastoma [39]. Consistently, our results show that the elevated expression of CHI3L1 in the transgenic mouse model for esophageal carcinoma is also associated with the upregulation of M2 macrophage markers. Furthermore, recombinant CHI3L1 also favors M2 marker gene expression upon LPS stimulation. Although the mechanism of CHI3L1-mediated M2 polarization remain to be further investigated, TGFβ signaling pathway may play an important role. TGF-β signaling activation polarizes macrophages toward a M2-like phenotype by up-regulating SNAIL [40], and it has been reported that CHI3L1 could activate TGFβ signaling pathway in hepatocellular carcinoma [41]. Therefore, the activation of TGFβ signaling pathway in TME may skews the macrophages toward a M2-like phenotype.

## Conclusion

In summary, our study revealed the upregulation of CHI3L1 in esophageal tumor tissues. Importantly, we showed a strong positive correlation between macrophage signatures and CHI3L1 expression level, indicating that CHI3L1 overexpression may favor macrophage recruitment in esophageal tumor tissues. Future studies are needed to further delineate the mechanisms of CHI3L1-mediated macrophage recruitment and polarization in tumor tissues in order to provide novel insights for therapeutic intervention.

## Highlights

- Chitinase-3 like-protein-1 (CHI3L1) is upregulated in esophagus cancers;
- CHI3L1 upregulation is correlated with macrophage signature genes;
- CHI3L1 is upregulated in esophagus dysplasia tissues in a transgenic mouse model;
- Recombinant CHI3L1 treatment regulates macrophage polarization gene expression.
